# Prevalence of E/A Wave Fusion and A Wave Truncation in DDD Pacemaker Patients with Complete AV Block under Nominal AV Intervals

**DOI:** 10.1371/journal.pone.0116075

**Published:** 2015-02-23

**Authors:** Wolfram C. Poller, Henryk Dreger, Marius Schwerg, Christoph Melzer

**Affiliations:** Department of Cardiology and Angiology, Charité University Hospital, Berlin, Germany; Scuola Superiore Sant'Anna, ITALY

## Abstract

**Aims:**

Optimization of the AV-interval (AVI) in DDD pacemakers improves cardiac hemodynamics and reduces pacemaker syndromes. Manual optimization is typically not performed in clinical routine. In the present study we analyze the prevalence of E/A wave fusion and A wave truncation under resting conditions in 160 patients with complete AV block (AVB) under the pre-programmed AVI. We manually optimized sub-optimal AVI.

**Methods:**

We analyzed 160 pacemaker patients with complete AVB, both in sinus rhythm (AV-sense; n = 129) and under atrial pacing (AV-pace; n = 31). Using Doppler analyses of the transmitral inflow we classified the nominal AVI as: a) normal, b) too long (E/A wave fusion) or c) too short (A wave truncation). In patients with a sub-optimal AVI, we performed manual optimization according to the recommendations of the American Society of Echocardiography.

**Results:**

All AVB patients with atrial pacing exhibited a normal transmitral inflow under the nominal AV-pace intervals (100%). In contrast, 25 AVB patients in sinus rhythm showed E/A wave fusion under the pre-programmed AV-sense intervals (19.4%; 95% confidence interval (CI): 12.6–26.2%). A wave truncations were not observed in any patient. All patients with a complete E/A wave fusion achieved a normal transmitral inflow after AV-sense interval reduction (mean optimized AVI: 79.4 ± 13.6 ms).

**Conclusions:**

Given the rate of 19.4% (CI 12.6–26.2%) of patients with a too long nominal AV-sense interval, automatic algorithms may prove useful in improving cardiac hemodynamics, especially in the subgroup of atrially triggered pacemaker patients with AV node diseases.

## Introduction

Year by year, the number of patients treated with a dual chamber (DDD) pacemaker has continually increased. Since their introduction, the optimal programming of the AV interval (AVI) has remained a controversial issue. The primary purpose of AVI optimization is to increase diastolic filling time without generating diastolic mitral regurgitation [1, 2]. Various methods to optimize the AVI have been developed [1, 3, 4]. The most common and feasible method employs pulsed-wave Doppler analysis of the transmitral inflow during diastole to optimize the AVI [1, 3]. Although large-scale trials have failed until now to demonstrate reduction in morbidity and mortality [5–7], several studies have shown beneficial effects of an optimal AVI on cardiac hemodynamics, as well as a reduction of pacemaker syndromes [2, 6]. Currently, individualized optimization of the AVI is not generally performed in the clinical routine, chiefly owing to the time-consuming process of manual optimization and to the lack of guideline recommendations [8, 9]. In recent years, several mechanisms of automatic AVI optimization have been introduced—mainly for CRT devices. Although automatic AVI optimization has become established for CRT devices, DDD pacemakers with this feature are currently not available.

Given the technical possibility, the current study was designed to analyze whether there is a need for automatic AVI optimization algorithms in DDD pacemakers. Since the higher the ventricular pacing rate, the more important an optimal AVI, patients with AV node diseases could especially benefit from optimization. We accordingly analyzed transmitral inflow in patients with complete AV block under nominal AV intervals to determine the prevalence of E/A wave fusion and A wave truncation as signs of sub-optimal AVI. Patients in sinus rhythm and patients under atrial pacing were separately analyzed. In case of sub-optimal AVI, we performed manual optimization.

## Methods

### Study population

Within a period of 14 months, 800 patients were followed up in our outpatient pacemaker clinic. Among these, we identified 160 patients with a DDD pacemaker who exhibited a complete AVB with an intrinsic ventricular rate below 30/min resulting in 100% RV-Pacing. 129 of these patients were in sinus rhythm (AV-sense), while 31 patients were under atrial pacing (AV-pace). We performed echocardiography and optimization during routine pacemaker follow-up, in accordance with the relevant guidelines [9]. Table 1 shows the baseline characteristics of the study groups.

**Table 1:** Baseline characteristics of the study groups (patients with complete AVB).

	all	AV-sense	AV-pace
n (%)	160 (100.0%)	129 (80.6%)	31 (19.4%)
males, n (%)	98 (61.3%)	78 (60.5%)	20 (64.5%)
age (years)	72.0 ± 14.3	71.9 ± 14.3	72.6 ± 14.4
LV ejection fraction (%)	52.9 ± 8.4	52.4 ± 8.6	55.2 ± 6.9
DDD implanted (years)	12.2 ± 11.6	11.7 ± 10.8	14.0 ± 14.3
Hypertension, n (%)	94 (58.8%)	76 (60.3%)	18 (61.3%)
CAD, n (%)	37 (23.1%)	29 (22.5%)	8 (25.8%)
Diabetes mellitus, n (%)	40 (25.0%)	32 (24.8%)	8 (25.8%)
Medtronic, n (%)	80 (50.0%)	66 (51.2%)	14 (45.2%)
Biotronik, n (%)	80 (50.0%)	63 (48.8%)	17 (54.8%)

When appropriate, data are given as mean ± standard deviation. Abbr.: AV-sense: patients in sinus rhythm; AV-pace: patients under atrial pacing

### Ethics statement

The study is in agreement with the Declaration of Helsinki. All procedures were performed as recommended by the guidelines and during the routine pacemaker follow-up. Data were analyzed anonymously. Patients gave written informed consent and the study is approved by the ethics committee of the Charité Universitätsmedizin Berlin.

### Echocardiography

**Fig 1:**
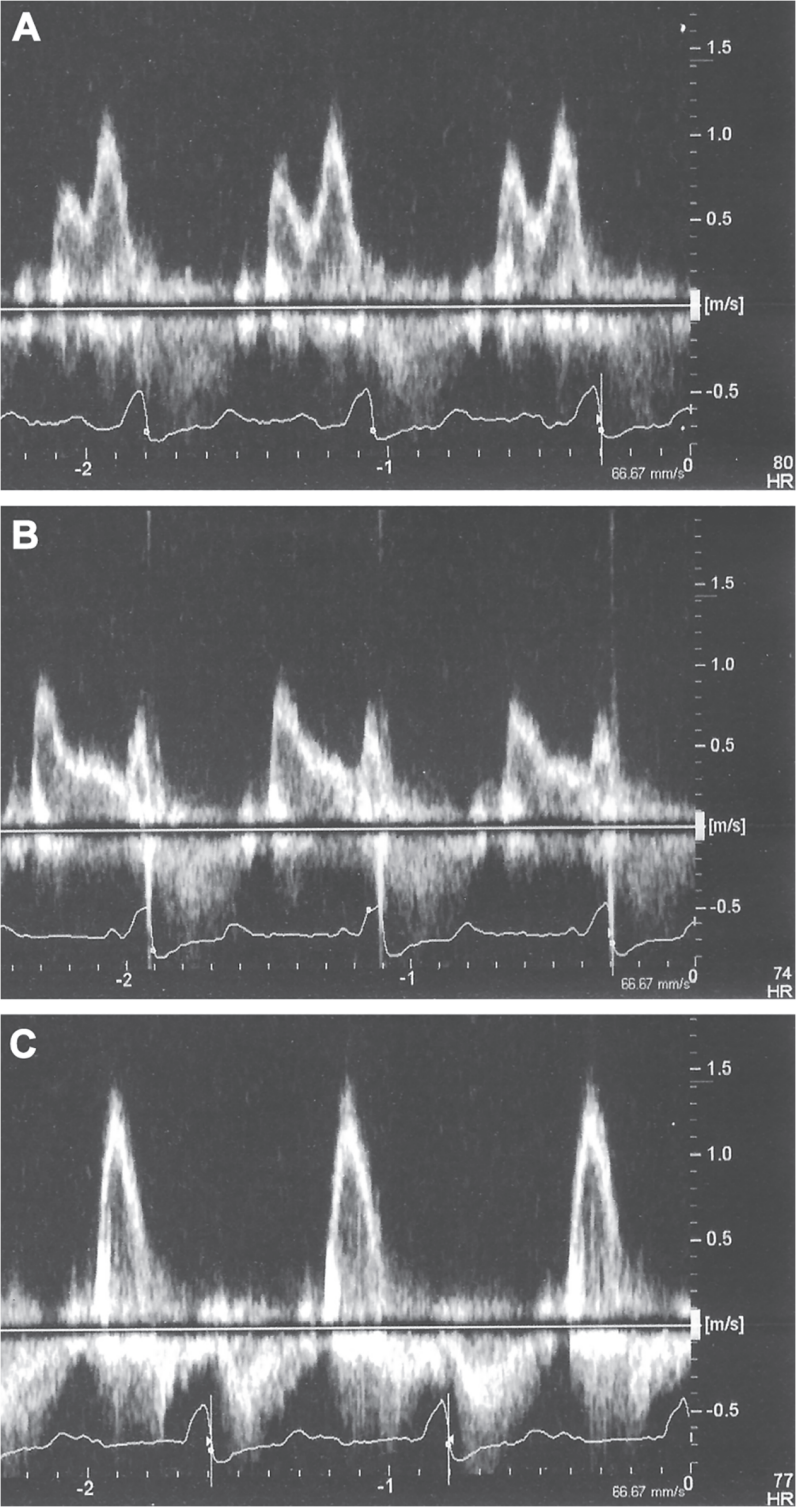
The AVI was classified as normal, too long or too short. To demonstrate the three categories of transmitral inflow pattern, one patient was analyzed with an optimal, a too short and a too long AV-sense interval. A) A normal AVI (135 ms) presents with separated E- and A waves, the A wave maximized in size and length and no diastolic mitral regurgitation. B) When the AVI is programmed too short (70 ms) an A wave truncation occurs (short, small and abruptly terminating A wave). C) A too long AVI (250 ms) presents with a complete E/A fusion. Such E/A fusion occurred in 19.4% (CI 12.6–26.2%) of the analyzed patients in sinus rhythm.

**Fig 2:**
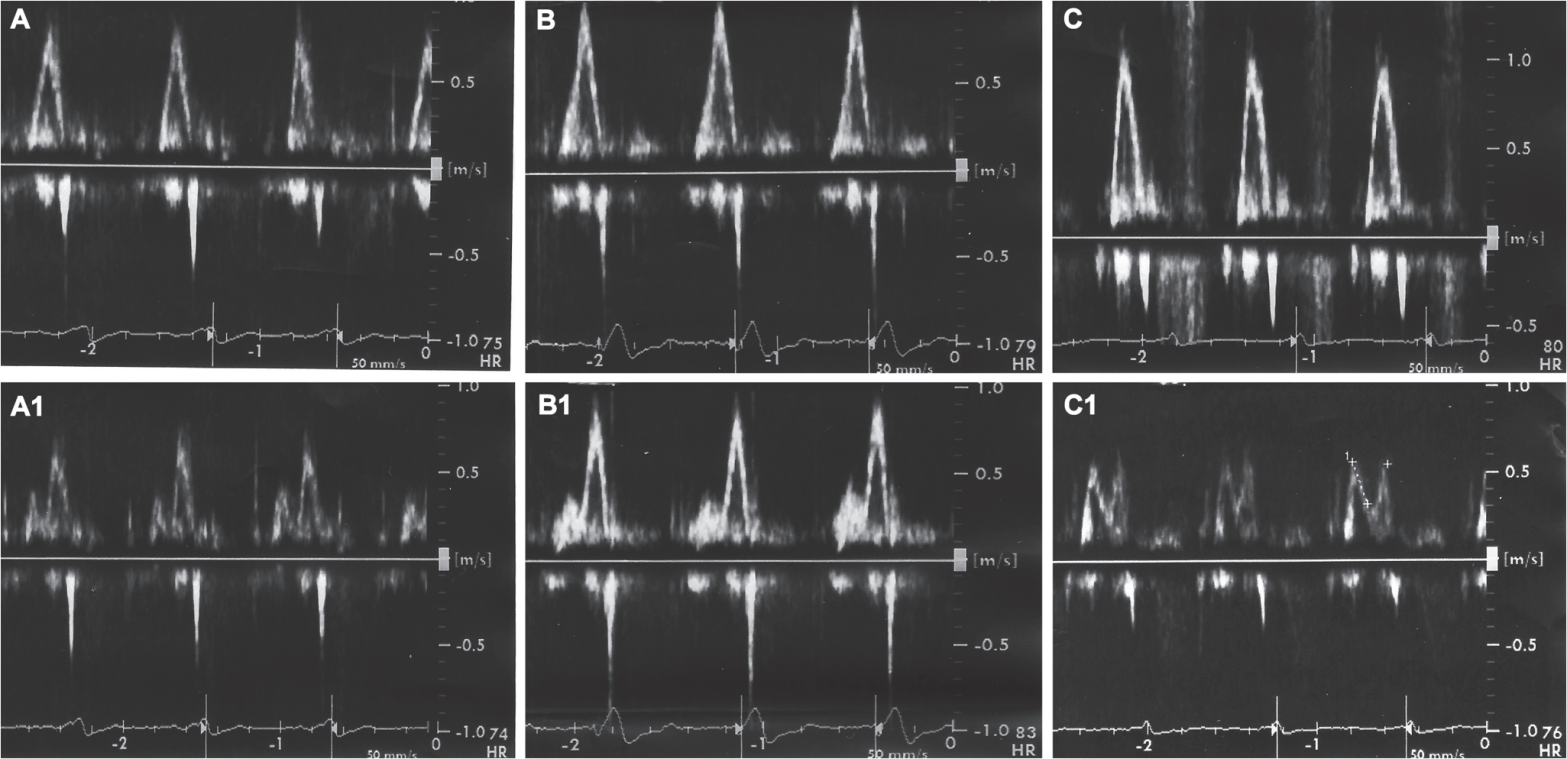
AVI reduction resolves E/A fusion. Transmitral PW Doppler recordings of three exemplary patients with E/A fusion under the nominal AV-sense intervals (125 ms in A, B, C). With AV-sense interval reduction to 75–100 ms (A1, B1, C1) the fusions resolve and E and A waves separate, indicating an improved transmitral inflow. The presented patients are representative for the 19.4% (CI 12.6–26.2%) of patients in sinus rhythm with a too long nominal AV-sense interval.

We examined all patients on a Vivid S6 Ultrasound System (GE Medical Systems, Norway). Initially, the pacemaker routine follow-up was performed in a lying position, which enabled patients to calm and to drop to their resting heart rate. To classify the nominal AVI as: a) normal, b) too long, or c) too short, we analyzed the transmitral inflow by pulsed-wave (PW-) Doppler, with positioning above the tips of the mitral valve leaflets. Three consecutive heart cycles were recorded with a sweep speed of 50–75 mm/s. Wall filter was optimized to detect diastolic mitral regurgitation according to current guidelines. A 3-lead ECG was superimposed on the Doppler recordings. A normal AVI is given when E and A waves are separated, the A wave is maximized in size and length and diastolic mitral regurgitation is absent (Fig. 1, A). A too long AVI was defined as complete fusion of E and A wave, i.e. both waves are not discriminable, and/or diastolic mitral regurgitation is present (Fig. 1, C). A too short AVI is indicated by a non-maximized A wave or an A wave truncation (Fig. 1, B). To determine whether the A wave was maximized in length and velocity, we programmed a long AVI (e.g., 180 ms) and recorded the transmitral PW Doppler signal. If the A wave did not increase compared to baseline, the A wave was defined normal [1, 3]. In case of an abnormal AVI, we performed manual optimization. Since we did not identify any patients with a too short AVI, only those 25 patients with E/A wave fusion underwent optimization. The AVI was accordingly reduced in 20-ms steps until fusion resolved and E and A wave fulfilled the criteria of a normal AVI, or until the minimum programmable AVI was reached (Fig. 2). We determined left ventricular ejection fraction (LVEF) using Simpson`s biplane approach. To exclude interobserver variability, one physician performed all examinations during presentation in the outpatient pacemaker clinic.

### Pacemakers

All patients had dual chamber pacemakers with a right atrial and a right ventricular electrode implanted according to current guidelines [9]. Devices were manufactured by Biotronik (Germany) (n = 80; 50.0%) and Medtronic (USA) (n = 80; 50.0%). In the Medtronic devices (Relia, Kappa, Adapta, Ensura, and Sigma) the nominal AV intervals are fixed at: AV-sense: 120 ms and AV-pace: 150 ms. In Biotronik pacemakers (Philos, Effecta, and Axios) the rate-adaptive mode is pre-activated, leading to an AV-sense interval of: 135 ms (60 bpm); 125 ms (80 bpm); 115 ms (100 bpm); 105 ms (120 bpm); 95 ms (140 bpm). The AV-pace interval is: 180 ms (60 bpm); 170 ms (80 bpm); 160 ms (100 bpm); 150 ms (120 bpm); 140 ms (140 bpm). We analyzed transmitral inflow only at the resting heart rate (Table 2).

**Table 2:** Transmitral inflow under nominal AV intervals and results of optimization.

	all	AV-sense	AV-pace
n (%)	160 (100.0%)	129 (80.6%)	31 (19.4%)
Heart rate (bpm)	72.6 ± 10.2	75.5 ± 9.1	60.6 ± 4.4 *
Nominal AVI (ms)	132.2 ± 18.8	123.9 ± 5.7	166.5 ± 14.9 *
AVI normal	135 (84.4%)	104 (80.6%)	31 (100%) *
AVI too long	25 (15.6%)	25 (19.4%)	0 (0%) *
AVI too short	0 (0.0%)	0 (0.0%)	0 (0%)
Optimization successful	-	25 (100%)	-
Mean optimized AVI (ms)	-	79.4 ± 13.6	-

When appropriate, data are given as mean ± standard deviation.

* *p* < 0.05 vs. AV-sense

Abbr.: AV-sense: patients in sinus rhythm; AV-pace: patients under atrial pacing

The atrial and ventricular lead positions were evaluated from post-OP chest X-rays and patients were grouped depending on RA and RV lead position: 1. RA-lead in the RA-appendage (RAA) and RV-lead in the apex: 80 patients (50.0%); 2. RA-lead in the RAA and RV-lead at the septum: 71 patients (44.4%); 3. RA-lead outside the RAA (mostly lateral wall) and RV-lead in the apex: 9 patients (5.6%); 4. RA-lead outside the RAA and RV-lead at the septum: 0 patients (0%). In the subgroup of patients with a too long nominal AV-sense interval, the distribution was similar (RAA-Apex: 13 patients (54.2%); RAA-Septum: 11 patients 45.8%). In the analyzed patients, 47 unipolar/passive fixation leads were used. All of them were located in the right ventricular apex. The other leads were bipolar and active fixating.

### Statistical analyses

When appropriate, data are expressed as mean ± standard deviation (SD). We conducted statistical tests using SigmaStat (SigmaStat 3.0, SPSS, Inc.). To calculate significance we performed t-tests, z-tests, and Mann-Whitney Rank Sum Tests. An error probability of p < 0.05 was considered statistically significant. We calculated 95% confidence intervals.

## Results

The average age of the analyzed patients was 72.0 ± 14.3 years. There were more male than female patients included (61.3% vs. 38.7%). The mean ejection fraction (LVEF) was 52.9 ± 8.4%. We identified 9 patients with indication for an implantable cardioverter/defibrillator (ICD) for primary prevention (LVEF below 35%). However, all patients had refused an ICD upgrade. The mean time since pacemaker implantation was 12.2 ± 11.6 years (Table 1). Among the analyzed patients, we observed no device-related problems during follow-up. The average examination time for the echocardiographic analyses of ejection fraction and Doppler parameters during routine follow-up was 3.0 minutes. In patients requiring AVI optimization, the examination time was 7.3 minutes. All patients had a sufficient acoustic window and were successfully analyzed.

Among 160 pacemaker patients with complete AVB, 80.6% were in sinus rhythm (n = 129), while 19.4% were atrially paced (n = 31) (Table 2). In patients under AV-sequential pacing, we observed neither E/A wave fusions nor A wave truncations, indicating that the nominal AV-pace intervals are adequate at rest. The mean AV-pace interval was 150 ms in Medtronic devices and 180 ms in Biotronik devices. The mean heart rate was 60.6 ± 4.4 bpm (Table 2).

Importantly, 19.4% (CI 12.6–26.2%) of the patients in sinus rhythm exhibited E/A wave fusion at rest, which indicated a too long AV-sense interval (Table 2). We observed no A wave truncations. The mean heart rate was 75.5 ± 9.1 bpm. The mean AV-sense interval was 123.9 ± 5.7 ms (Table 2).

In patients with E/A wave fusion, the heart rate was slightly higher compared to the rest of the patients in sinus rhythm (78.7 ± 6.7 bpm vs. 74.7 ± 9.4 bpm; p = 0.047). The nominal AV-sense intervals were not significantly different 122.2 ± 4.9 ms in patients with E/A fusion vs. 124.3 ± 5.9 ms in patients without E/A fusion; p = 0.096).

In all 25 patients with an E/A wave fusion at baseline, normal transmitral inflow was achieved by echo-guided reduction of the AV-sense interval (3 examples in Fig. 2). In most of the patients, considerable AVI reduction was necessary to normalize the LV filling profile (mean optimized AV-sense interval: 79.4 ± 13.6 ms) (Table 2). Importantly, all patients demonstrated a constant heart rate during optimization (78.7 ± 6.7 bpm), with the result that rate changes did not influence the E/A profile.

In a subgroup analysis we compared all patients in sinus rhythm with Medtronic devices, in which the AV-sense interval was fixed at 120 ms (n = 66), with patients with Biotronik devices with a rate-adaptive AV-sense interval (n = 63). Based on resting heart rate at presentation, the mean rate-adaptive AVI in the Biotronik group was slightly longer (Biotronik group: 128.0 ± 5.8 ms vs. Medtronic group: 120.0 ± 0.0 ms; p<0.05). As expected from the longer mean AV-sense interval in the Biotronik group, there were significantly more patients with an E/A wave fusion in the Biotronik group (Medtronic group: 12.1% vs. Biotronik group: 26.9%; p = 0.03).

## Discussion

The present analysis identified a relevant proportion of patients with complete AVB who exhibited sub-optimal LV filling under nominal AV intervals. Whereas the nominal AV-pace intervals were adequate in all patients, the AV-sense intervals were too long in 19.4% (CI 12.6–26.2%) of the patients. Importantly, manual AVI optimization led to normalized LV filling in all patients with baseline E/A fusion.

Our results seem to be representative for the majority of AVB patients, since a routine optimization of the AVI is performed only in individual cases [9, 10]. The echocardiographic methodology that we applied to analyze the AVI is well accepted and feasible in clinical routine [1, 3, 10]. The observation that a mean pre-programmed AV-sense interval of 123.9 ± 5.7 ms is too long for some patients concurs well with several other studies that have reported “optimal” AV delays for atrial triggering (e.g., Kindermann et al. AVI: 88 ± 35 ms and Knorre et al. AVI: 100.5 ± 27.8 ms). However, a small number of other studies considered a longer AVI optimal, e.g. Janosik et al.: 144 ± 48 ms for atrial triggering [10–12]. The optimal AVI is a highly individual characteristic of each patient. The nominal AV-sense intervals are shorter because the atrial lead detects an intrinsic electric signal that is already running across the atrial myocardium. The AV-pace intervals are longer because, in the case of atrial stimulation, the pacing stimulus takes place at the beginning of atrial activation. The position of the atrial and ventricular leads, the atrial size, intra-atrial conduction, and other cellular and subcellular mechanisms are contributing parameters [13]. The slightly higher resting heart rate in patients with E/A wave fusion also seems to contribute. However, a mean heart rate of 78.7 ± 6.7 bpm should not physiologically cause complete E/A wave fusion and represents a permanent condition in these patients (resting heart rate measured in a lying position after calming down). In patients under atrial pacing, the significantly lower heart rate of 60.6 ± 4.4 bpm certainly contributes to the normal E/A profile. The effect of the R-Mode under physical activity cannot be predicted from the current data.

Cannon waves result from a too short AVI, while a too long AVI causes diastolic mitral regurgitation. Pacemaker syndromes occur in more than one quarter of VVI-paced AVB patients in sinus rhythm [9]. Sub-optimal AVI programming can result in a 10% to 15% decline in cardiac output [14]. Supporting this observation, many trials have shown acute hemodynamic benefits of AV optimization [2, 6, 15, 16]. Despite all data on acute hemodynamic improvement, there is currently no guideline demand for routine AVI optimization in DDD pacemaker patients [9]. The reason is that, until now, large trials have not brought evidence for incremental long-term benefit in reduction of morbidity or mortality [5–7]. Despite the broad spectrum of invasive and non-invasive optimization methods, no single method is currently recommended for routine clinical practice, since evidence from randomized controlled trials is lacking [9, 10]. Furthermore, all manual methods require operating experience, time, and other scarce resources. In this situation of beneficial hemodynamic effects and reduction of pacemaker syndromes on the one hand, but the effort of manual optimization required on the other hand, an automatic optimization algorithm could well be helpful.

In recent years, several automatic optimization mechanisms have been developed for CRT devices. These mechanisms, intended to avoid the time-consuming process of manual Doppler optimization, are automatic electrocardiogram- or peak endocardial acceleration-based algorithms to optimize AV- and VV delays as evaluated in the SMART-AV Study [5], the Freedom Study [17], the CLEAR Study [18], and the adaptive CRT trial [7]. Available AV delay optimization mechanisms include the SmartDelay algorithm, which is implemented in Boston Scientific CRT devices [5]. It is based on early studies that analyzed hemodynamic changes measured by LV dP/dt and pulse pressure during CRT at various combinations of AV delays [15, 16]. SmartDelay is ECG-based and calculates sensed and paced AV delays from the intracardiac electrograms. These delays are added to the surface QRS duration to estimate the optimal AVI. A competing automatic algorithm is named QuickOpt and was introduced by St. Jude Medical [17]. It also optimizes the AV delay on the basis of intracardiac electrograms. QuickOpt uses the right atrial intracardiac electrogram to measure the intra-atrial conduction delay, which is the major variable of the optimal individual AVI [13]. Based on this interval, an offset is added and programmed as optimal AVI [13]. In contrast to SmartDelay, this method is in principle applicable for patients with complete AVB. A completely different algorithm is called SonR by Sorin Biomedica. It uses a microaccelerometer located in an intracardiac electrode (SonRTip) to determine peak endocardial acceleration (PEA). The PEA has been shown to correlate well with maximum contractility [18, 19]. The CLEAR Study has compared AV and VV optimization by PEA with standard care and has observed a higher response rate in a CRT cohort [18]. This algorithm does not require intrinsic conduction and would therefore be useful for AVB patients. The last automatic algorithm currently on the market is CardioSync implemented in the AdaptiveCRT algorithm of Medtronic [7]. This algorithm measures the patient’s paced and sensed AV intervals and the waveform widths of the P wave and QRS complex and adjusts the AVI accordingly. Although some outcome studies have already been published and so far did not show incremental clinical benefits [5, 7, 17, 20], the final value and ranking of these various automatic algorithms are still controversial.

Given these promising new algorithms and the relevant proportion of patients with a too long nominal AV-sense interval that is currently not optimized in clinical routine, it seems beneficial to adapt automatic algorithms for use in DDD pacemakers. Future studies are required to determine whether implementation of such mechanisms will ultimately improve symptoms or outcome of AVB patients.

### Limitations

The current study analyzed the AVI with focus on diastolic function and with one method (transmitral PW Doppler). In this study we did not analyze the clinical outcome of optimization. However, more extensive patient cohorts and follow-up periods would be necessary to determine the clinical benefit of optimization. We included only patients with Medtronic and Biotronik pacemakers. However, the other two major companies St. Jude and Boston Scientific have even longer pre-programmed AV-sense intervals (150 ms), suggesting that the percentage of too long nominal AV-sense intervals might be even higher in these patients. Transmitral inflow was classified as normal, too long, or too short. While an A wave truncation is an accepted sign of a too short AVI and an E/A wave fusion at rest clearly indicates a too long AVI, the definition of a normal or even optimal AVI is more difficult. There are a number of concepts for further optimizing the AVI (e.g., Ritter’s concept) that are beyond the scope of this study. Here, we describe only the prevalence of clearly sub-optimal nominal AV intervals and the possibility of manually optimizing them. Based on this study, we have posited the hypothesis that automatic algorithms for DDD pacemakers could well be helpful. This hypothesis must be proven. Finally, the AVI was analyzed with subjects only at rest.

### Conclusions

Not all pacemaker patients with complete AV block have normal LV-filling profiles under the pre-programmed AV intervals. Among patients in sinus rhythm, 19.4% (CI 12.6–26.2%) exhibit signs of a clearly too long nominal AV-sense interval. This cohort of patients would benefit from optimization, which is frequently not performed in clinical routine. Automatic AVI optimization algorithms could consequently support an enhancement in AV hemodynamics, especially in this subgroup of pacemaker patients.
